# Role of the *Streptococcus suis* serotype 2 capsular polysaccharide in the interactions with dendritic cells is strain-dependent but remains critical for virulence

**DOI:** 10.1371/journal.pone.0200453

**Published:** 2018-07-12

**Authors:** Jean-Philippe Auger, Dominic Dolbec, David Roy, Mariela Segura, Marcelo Gottschalk

**Affiliations:** Research Group on Infectious Diseases in Production Animals (GREMIP) and Swine and Poultry Infectious Diseases Research Center (CRIPA), Department of Pathology and Microbiology, Faculty of Veterinary Medicine, University of Montreal, Saint-Hyacinthe, Quebec, Canada; Instituto Butantan, BRAZIL

## Abstract

*Streptococcus suis* serotype 2 is an important porcine bacterial pathogen and zoonotic agent responsible for sudden death, septic shock, and meningitis. However, serotype 2 strains are heterogeneous, composed of a multitude of sequence types (STs) whose distribution greatly varies worldwide. Of the virulence factors presently described for *S*. *suis*, the capsular polysaccharide (CPS) is a critical factor implicated in a multitude of functions, including in impairment of phagocytosis and innate immune cell activation by masking underlying bacterial components. However, these roles have been described using Eurasian ST1 and ST7 strains, which greatly differ from North American ST25 strains. Consequently, the capacity of the CPS to mask surface antigens and putative virulence factors in non-Eurasian strains remains unknown. Herein, the role of the *S*. *suis* serotype 2 CPS of a prototype intermediate virulent North American ST25 strain, in comparison with that of a virulent European ST1 strain, with regards to interactions with dendritic cells, as well as virulence during the systemic phase of infection, was evaluated. Results demonstrated that the CPS remains critical for virulence and development of clinical disease regardless of strain background, due to its requirement for survival in blood. However, its role in the interactions with dendritic cells is strain-dependent. Consequently, certain key characteristics associated with the CPS are not necessarily applicable to all *S*. *suis* serotype 2 strains. This indicates that though certain factors may be important for *S*. *suis* serotype 2 virulence, strain background could be as determining, reiterating the need in using strains from varying backgrounds in order to better characterize the *S*. *suis* pathogenesis.

## Introduction

*Streptococcus suis* is an important bacterial pathogen of young piglets and a zoonotic agent causing sudden death (pigs), septic shock (humans), and meningitis (both species) [1]. Classification is based on serotyping defined by the capsular polysaccharide (CPS) present or its genes [1]. Worldwide, serotype 2 is the most widespread and virulent, responsible for the majority of porcine and human cases of infection [2]. However, an extensive heterogeneity exists within serotype 2 strains, both genotypically and phenotypically, which can be grouped into a multitude of sequence types (STs), as determined by multilocus sequence typing, and whose distribution greatly varies [2]. Furthermore, experimental mouse and pig models of infection have been used to determine virulence of the most important STs (ST1, ST7, and ST25) [3–7]. While the ST7 strain responsible for the Chinese human outbreaks is highly virulent, European ST1 strains are virulent; by contrast, ST25 strains, typical in North America, are of intermediate virulence [3, 8]. Albeit these important differences, studies on the pathogenesis of the infection have almost exclusively used Eurasian strains, even though ST25 strains account for nearly 50% of serotype 2 isolates recovered from diseased pigs in North America [9].

Though a variety of virulence factors have been described for *S*. *suis*, the CPS, which is a critical factor whose composition and structure is identical for all serotype 2 strains, is implicated in a multitude of functions [10, 11]. Of these, resistance to phagocytosis by innate immune cells [12–16] and masking of surface components responsible for host cell activation [16, 17] are the most important. Studies using experimental animal infection models have demonstrated that the CPS is required for survival in blood [18, 19]. Alongside, it was recently demonstrated that *S*. *suis* can modulate presence of its CPS within the host [20]. However, these roles have been described using Eurasian ST1 and ST7 strains, which greatly differ from their North American counterparts.

The innate immune host response is composed of numerous cell types of which phagocytes play a central role in the *S*. *suis* pathogenesis [10, 11]. Of these, dendritic cells (DCs) are key players involved in phagocytosis and cytokine production [21]. Indeed, DCs have been previously described as playing an important role in the *S*. *suis* pathogenesis by contributing to the exacerbated inflammatory host response [16, 22]. Nevertheless, the interactions between DCs and *S*. *suis* serotype 2 have been mostly studied using Eurasian strains.

Though the CPS is antigenically identical for all *S*. *suis* serotype 2 strains, its capacity to mask surface antigens and putative virulence factors in non-Eurasian strains remains unknown. Consequently, the objective of this study was to evaluate the role of the *S*. *suis* serotype 2 CPS of a prototype intermediate virulent North American ST25 strain, in comparison with that of a virulent European ST1 strain, with regards to interactions with DCs, as well as virulence during the systemic phase of infection. Results obtained demonstrate that while the CPS is required for survival in blood and remains critical for virulence and development of clinical disease regardless of strain origin, its role in the interactions with DCs (resistance to phagocytosis and interference of cytokine production) is strain-dependent.

## Materials and methods

### Ethics statement

This study was carried out in accordance with the recommendations of the guidelines and policies of the Canadian Council on Animal Care and the principles set forth in the Guide for the Care and Use of Laboratory Animals. The protocols and procedures were approved by the Animal Welfare Committee of the University of Montreal (permit number rech-1570).

### Bacterial strains and growth conditions

The well-characterized and highly encapsulated wild-type *S*. *suis* serotype 2 strains and non-encapsulated isogenic mutants used in this study are listed in Table 1. *S*. *suis* strains were cultured in Todd Hewitt broth (THB; Becton Dickinson, Mississauga, ON). For *in vitro* cell culture assays, bacteria were prepared as previously described [16] and resuspended in cell culture medium. For experimental infections, early stationary phase bacteria were washed twice in phosphate-buffered saline pH 7.4 and resuspended in THB [4, 23, 24]. Bacterial cultures were appropriately diluted and plated on THB agar (THA) to accurately determine bacterial concentrations. The *Escherichia coli* strain and different plasmids used in this study are also listed in Table 1. When needed, antibiotics (Sigma-Aldrich, Oakville, ON) were added to the media at the following concentrations: for *S*. *suis*, spectinomycin at 100 μg/mL; for *E*. *coli*, kanamycin and spectinomycin at 50 μg/mL and ampicillin at 100 μg/mL.

**Table 1:** List of strains and plasmids used in this study.

Strain or plasmid	Characteristics	Reference
***Streptococcus suis***		
P1/7	Virulent European ST1 strain isolated from a case of pig meningitis in the United Kingdom	[25]
P1/7Δ*cpsF*	Non-encapsulated isogenic mutant derived from P1/7; in frame deletion of *cpsF* gene	[16]
P1/7Δ*neuC*	Non-encapsulated isogenic mutant derived from P1/7; in frame deletion of *neuC* gene	[18]
89–1591	Intermediate virulent North American ST25 strain isolated from a case of pig sepsis in Canada	[26]
89–1591Δ*cpsF*	Non-encapsulated isogenic mutant derived from 89–1591; in frame deletion of *cpsF* gene	This study
89–1591Δ*neuC*	Non-encapsulated isogenic mutant derived from 89–1591; in frame deletion of *neuC* gene	This study
***Escherichia coli***		
TOP10	F^-^ mrcA Δ(mrr-hsdRMS-mcrBC) φ80 lacZΔM15 ΔlacX74 recA1 araD139 Δ(ara-leu) 7697 galU galK rpsL (Str^R^) endA1 nupG	Invitrogen
**Plasmids**		
pCR2.1	Ap^r^, Km^r^, pUC *ori*, *lac*ZΔM15	Invitrogen
pSET4s	Spc^r^, pUC *ori*, thermosensitive pG+host3 *ori*, *lac*ZΔM15	[27]
p4Δ*cpsF*	pSET4s carrying the construct for *cpsF* allelic replacement	This study
p4Δ*neuC*	pSET4s carrying the construct for *neuC* allelic replacement	This study

### DNA manipulations

As previously described [28], *S*. *suis* genomic DNA was extracted using InstaGene Matrix solution (BioRad Laboratories, Hercules, CA). Mini-preparations of recombinant plasmids were carried out using the QIAprep Spin Miniprep Kit (Qiagen, Valencia, CA). Restriction enzymes and DNA-modifying enzymes (Fisher Scientific, Ottawa, ON) were used according to the manufacturer’s recommendations. Oligonucleotide primers (Table 2) were obtained from Integrated DNA Technologies (Coralville, IA) and PCRs carried out with the iProof proofreading DNA polymerase (BioRad Laboratories, Mississauga, ON) or the Taq DNA polymerase (Qiagen). Amplification products were purified using the QIAquick PCR Purification Kit (Qiagen) and sequenced using an ABI 310 Automated DNA Sequencer and ABI PRISM Dye Terminator Cycle Sequencing Kit (Applied Biosystems, Carlsbad, CA).

**Table 2:** List of oligonucleotide primers used in this study.

Name	Sequence (5’– 3’)
**PCR**	
*cpsF*-1	CCA GCA AAG TAT GGT GGT TTC G
*cpsF*-2	GCG CAC CAA CTT CTC TTA ATG C
*cpsF*-3	CTT AGT CAC TCC GAA CTC ACC G
*cpsF*-4	CCA CGC CAG ATT CAA TGA GC
*cpsF*-5	AGA CGG TCA TGA ATG GCT ACG
*cpsF*-6	GAG GGA GGT GTA GAC TTC TGC TCC AGC ATG
*cpsF*-7	CAT GCT GGA GCA GAA GTC TAC ACC TCC CTC
*cpsF*-8	CAT CAG AAT GAT GCC AAA CAG G
*neuC-*1	TGC CCG TTT ATA AGA TTC CAT C
*neuC-*2	TGA GTT GCT CTG TCA AGG TC
*neuC-*3	TGA TTG AAG TGC CCT CAT TAC
*neuC-*4	TAA ACC TTT TGA TCC TGA CCG
*neuC-*5	TGA AAA GCA CTT TAC TCT GGA C
*neuC-*6	CCT TGT AAA GCA GAA TCA GGT TGA TGC ATG GCT GTC ACT AC
*neuC-*7	GTA GTG ACA GCC ATG CAT CAA CCT GAT TCT GCT TTA CAA GG
*neuC-*8	ATG TTC CAC AAT GGC ACC C
**Quantitative real-time PCR**	
*Atp5b*	F: ACC AGC CCA CCC TAG CCA CC
	R: TGC AGG GGC AGG GTC AGT CA
*Gapdh*	F: CCC GTA GAC AAA ATG GTG AAG
	R: GAC TGT GCC GTT GAA TTT G
*Ifnb*	F: CCC AGT GCT GGA GCC ATT GT
	R: CCC TAT GGA GAT GAC GGA GA

### Construction of the 89–1591 non-encapsulated isogenic mutants 89-1591Δ*cpsF* and 89-1591Δ*neuC*

The P1/7 non-encapsulated isogenic mutants P1/7Δ*cpsF* and P1/7Δ*neuC* were constructed and characterized elsewhere [16, 18]. In this study, the DNA genome sequence of the *S*. *suis* wild-type strain 89–1591 was used for construction of its non-encapsulated isogenic mutants. Precise in-frame deletions of *cpsF* or *neuC* genes were constructed using splicing-by-overlap-extension PCRs as previously described [28, 29]. Overlapping PCR products were cloned into pCR2.1 (Invitrogen, Burlington, ON), extracted with EcoRI, recloned into the thermosensitive *E*. *coli*–*S*. *suis* shuttle plasmid pSET4s, and digested with the same enzyme, giving rise to the knockout vector p4Δ*cpsF* or p4Δ*neuC*. Electroporation of the wild-type strain 89–1591 and procedures for isolation of the mutants were previously described [27]. Allelic replacement was confirmed by PCR and DNA sequencing analyses. Amplification products were purified with the QIAgen PCR Purification Kit (Qiagen) and sequenced as described above. Growth of both mutants was similar to that of the wild-type strain (data not shown).

### Bacterial surface hydrophobicity assay

Relative surface hydrophobicity of the *S*. *suis* wild-type strains and non-encapsulated mutants was determined by measuring adsorption to *n*-hexadecane as previously described [30].

### Transmission electron microscopy

Unless otherwise indicated, chemicals were purchased from Sigma-Aldrich. Transmission electron microscopy was carried out as previously described [31, 32]. Briefly, bacteria were grown to mid-logarithmic phase and washed in 0.1 M cacodylate buffer pH 7.3 (Canemco & Marivac, Canton de Gore, QC) containing 2.5% glutaraldehyde and 0.05% ruthenium red. Ferritin was then added to a final concentration of 1 mg/mL and incubated for 30 min at room temperature. Cells were then immobilized in 3% agar in 0.1 M cacodylate buffer pH 7.3, washed five times in cacodylate buffer containing 0.05% ruthenium red, and fixed in 2% osmium tetroxide for 2 h at room temperature. Afterwards, samples were washed with water every 20 min for 2 h to remove osmium tetroxide and dehydrated in an increasing graded series of acetone. Specimens were then washed twice in propylene oxide and embedded in Spurr low-viscosity resin (Electron Microscopy Sciences, Hatfield, PA). Thin sections were post-stained with uranyl acetate and lead citrate and examined using a transmission electron microscope at 80 kV (Hitachi model HT7770, Chiyoda, Tokyo, Japan).

### Generation of bone marrow-derived dendritic cells

The femur and tibia from eight C57BL/6 mice (Jackson Research Laboratories, Bar Harbour, ME) were used to generate bone marrow-derived DCs as previously described [16]. Briefly, hematopoietic bone marrow stem cells were cultured in RPMI-1640 medium supplemented with 5% heat-inactivated fetal bovine serum, 10 mM HEPES, 2 mM L-glutamine, and 50 μM 2-mercaptoethanol (Gibco, Burlington, ON). Complete medium was complemented with 20% granulocyte macrophage-colony stimulating factor from mouse-transfected Ag8653 cells [33]. Cell purity was confirmed to be at least 85% CD11c^+^ by flow cytometry as previously described [16]. Prior to infection, cells were resuspended at 1 x 10^6^ cells/mL in complete medium and stimulated with the different strains of *S*. *suis* serotype 2 listed in Table 1 (1 x 10^6^ CFU/mL; initial multiplicity of infection [MOI] = 1). Conditions used were based on those previously published [16, 22].

### Internalization assay

Cells were infected with the different *S*. *suis* strains and phagocytosis was left to proceed for different times (0.5 to 4 h) at 37°C with 5% CO_2_. MOI and assay conditions were chosen based on previous studies regarding the kinetics of *S*. *suis* phagocytosis by DCs [16]. After incubation, penicillin G (5 mg/mL; Sigma-Aldrich) and gentamicin (100 mg/mL; Gibco) were added to the wells for 1 h to kill extracellular bacteria. Supernatant controls were taken in every test to confirm that extracellular bacteria were efficiently killed by the antibiotics. After antibiotic treatment, cells were washed three times and water added to lyse the cells. The number of CFU recovered per well was determined by plating viable intracellular bacteria on THA.

### Cytokine measurement

Supernatants were collected 16 h following infection with *S*. *suis*, time at which secreted cytokine levels are maximal in the absence of *S*. *suis*-induced DC cytotoxicity [16, 22]. Non-infected cells served as negative controls. Secreted levels of tumor necrosis factor (TNF), interleukin (IL)-6, IL-12p70, and C-X-C motif chemokine ligand (CXCL) 1 were quantified by sandwich ELISA using pair-matched antibodies from R&D Systems (Minneapolis, MN) according to the manufacturer’s recommendations.

### Determination of interferon-β mRNA expression by quantitative RT-PCR

Cell mRNA was extracted 6 h post-infection (p.i) according to the manufacturer’s instructions (Qiagen) and cDNA generated using the Quantitect cDNA Synthesis Kit (Qiagen) as previously described [32]. Incubation time was chosen based on maximal expression of interferon (IFN)-β by DCs following *S*. *suis* infection [32]. Real-time qPCR was performed on the CFX-96 Touch Rapid Thermal Cycler System (Bio-Rad) using 250 nM of primers (Integrated DNA technologies) and the SsoFast Evagreen Supermix Kit (Bio-Rad). Cycling conditions were 3 min of polymerase activation at 98°C, followed by 40 cycles at 98°C for 2 sec and 57°C for 5 sec. Melting curves were generated after each run to confirm presence of a single PCR product. The primer sequences used in this study are shown in Table 2 and were verified to have reaction efficiencies between 90% and 110%. The reference genes *Atp5b* and *Gapdh*, determined to be the most stably expressed using the algorithm geNorm, were used to normalize data. Fold changes in gene expression were calculated using the quantification cycle threshold (Cq) method using the CFX software manager v.3.0 (Bio-Rad). Samples from mock-infected cells served as calibrators. IFN-β was measured by RT-qPCR in order to compare with published results [32].

### Whole blood bactericidal (killing) assay

Blood was collected from twelve six- to ten-week-old female CD-1 mice (Charles River Laboratories, Wilmington, MA) and mixed with sodium heparin (Sigma-Aldrich). Leukocytes (9 x 10^6^ cells/mL on average) were transferred to a microtube containing 9 x 10^6^ CFU/mL of the different *S*. *suis* strains (MOI = 1) and incubated for 4 h, mixing every 20 min. Assay conditions were chosen based on the kinetics of *S*. *suis* killing by murine blood [4]. After incubation, cells were lysed by vortexing and vigorous pipetting and appropriate dilutions plated on THA to determine viable bacterial counts. Resistance to bacterial killing by blood leukocytes was compared to incubation of the different bacterial strains in plasma only (obtained by centrifuging whole blood at 1 800 x *g* for 10 min at 4°C). The percentage of bacteria killed was determined using the following formula: 1 - (bacteria in blood / bacteria in plasma) / 100%.

### *S*. *suis* virulence mouse model of infection

A well-standardized CD-1 mouse model of infection was used [18, 23]. These studies were carried out in strict accordance with the recommendations of and approved by the University of Montreal Animal Welfare Committee guidelines and policies, including euthanasia to minimize animal suffering through the use of humane endpoints, applied throughout this study when animals were seriously affected since mortality was not an endpoint measurement. No additional considerations or housing conditions were required. All staff members received the required animal handling training as administered by the University of Montreal Animal Welfare Committee. Forty-five six-week-old female CD-1 mice (Charles River Laboratories) were used for these experiments (15 mice per strain evaluated). Mice were inoculated with 5 x 10^7^ CFU via the intraperitoneal route and health and behavior monitored at least thrice daily until 72 h p.i. and twice thereafter until the end of the experiment (10 days p.i.) for the development of clinical signs of sepsis, such as depression, swollen eyes, rough hair coat, and lethargy. Mice were also monitored for the development of clinical signs of meningitis. Clinical scores were determined according to the grid approved by the University of Montreal Animal Welfare Committee (S1 Appendix) and required actions undertaken. Mice were immediately euthanized upon reaching endpoint criteria using CO_2_ followed by cervical dislocation. No mice died before meeting endpoint criteria and all surviving mice were euthanized as described above at the end of the experiment (10 days p.i.). Blood samples were collected from the caudal vein of surviving mice 24 h p.i. and plated as previously described [4].

### Statistical analyses

Normality of data was verified using the Shapiro-Wilk test. Accordingly, parametric (unpaired t-test) or non-parametric tests (Mann-Whitney rank sum test), where appropriate, were performed to evaluate statistical differences between groups. Log-rank (Mantel-Cox) tests were used to compare survival between wild-type-infected mice and those infected with the non-encapsulated strains. Each *in vitro* test was repeated in at least three independent experiments. *p* < 0.05 was considered as statistically significant.

## Results

### Deletion of *cpsF* or *neuC* genes involved in *S*. *suis* serotype 2 capsular polysaccharide biosynthesis results in non-encapsulation of a ST25 strain

**Fig 1:**
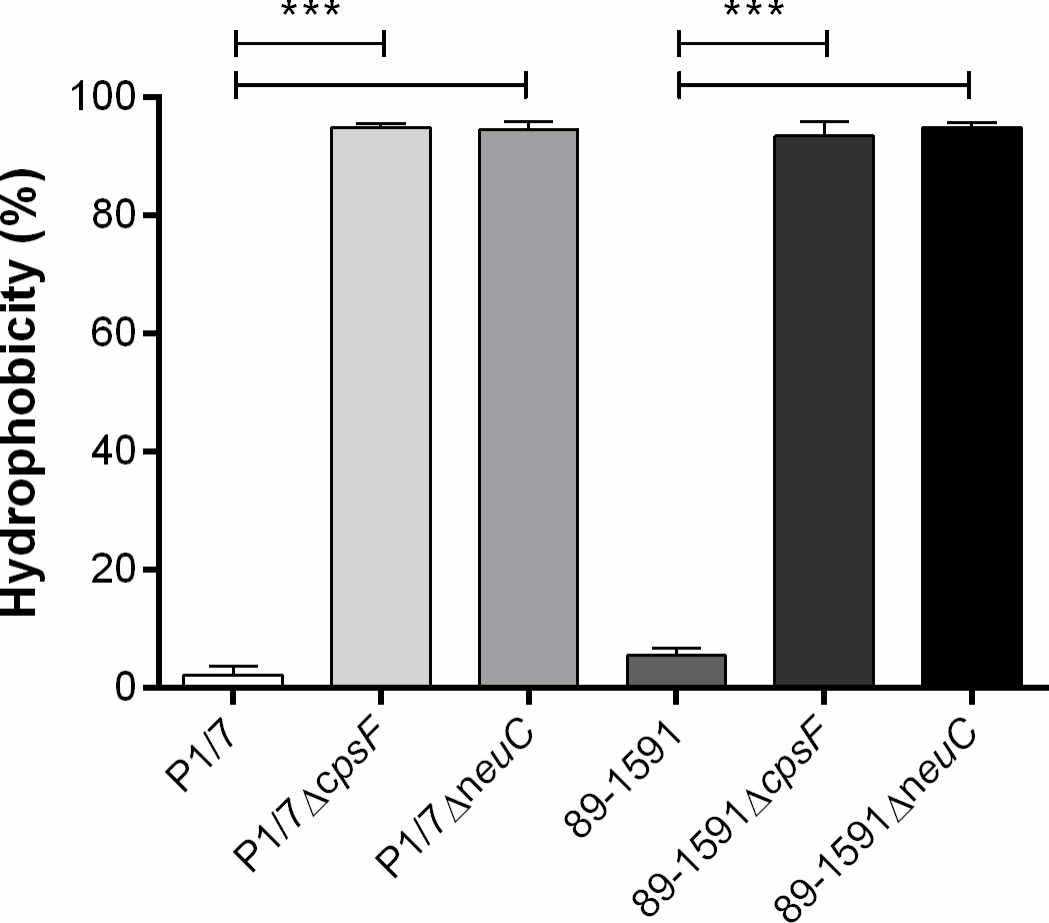
Absence of capsular polysaccharide is associated with increased surface hydrophobicity of *S*. *suis* serotype 2. Hydrophobicity of wild-type and mutant strains was determined using *n*-hexadecane. Data represent the mean ± SEM from three independent experiments. *** (*p* < 0.001) indicates a significant difference between wild-type strains (P1/7 or 89–1591) and their non-encapsulated mutants (Δ*cpsF* and Δ*neuC*).

Though deletion of various CPS biosynthesis genes, including those for the sialic acid side-chain, was previously described to result in absence of CPS in Eurasian ST1 and ST7 strains [16, 18, 19, 34], the effect of *cpsF* or *neuC* gene deletion on CPS expression in a ST25 strain was unknown. Surface hydrophobicity (an indicator of encapsulation) was similar between wild-type strains P1/7 (ST1) and 89–1591 (ST25) (Fig 1). Deletion of *cpsF* or *neuC* gene significantly increased surface hydrophobicity of all mutants (*p* < 0.001), which averaged 95% (Fig 1). These results confirm that deletion of *S*. *suis* serotype 2 CPS biosynthesis genes results in high surface hydrophobicity, regardless of strain background.

**Fig 2:**
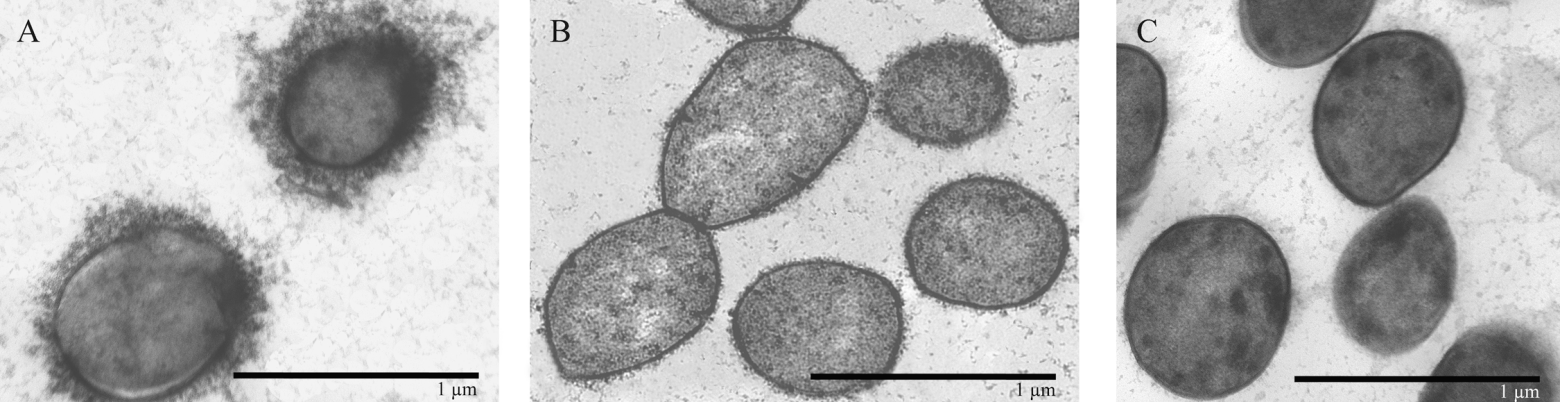
Deletion of genes involved in *S*. *suis* serotype 2 capsular polysaccharide biosynthesis (*cpsF* and *neuC*) results in non-encapsulation of the ST25 strain 89–1591. Transmission electron microscopy following labelling with polycationic ferritin of the ST25 wild-type strain 89–1591 (A) or its isogenic mutants 89–1591Δ*cpsF* (B) and 89–1591Δ*neuC* (C). Black bars = 1 μm.

Encapsulation of the ST25 strain 89–1591 and its *cpsF*- and *neuC*-deficient mutants was then evaluated by transmission electronic microscopy following labelling with polycationic ferritin, which binds negatively charged structures such as the *S*. *suis* serotype 2 CPS [31]. In accordance with previously published micrographs [26, 32], wild-type strain 89–1591 is well-encapsulated, presenting a thick layer of CPS at its surface as evidenced by the polycationic ferritin labelling (Fig 2A). This is similar to results obtained with the European ST1 strain P1/7 [18]. On the other hand, 89–1591 mutants deficient for either *cpsF* (Fig 2B) or *neuC* (Fig 2C) gene clearly lack polycationic ferritin marker at their surface, indicating a lack of CPS. Consequently, these results confirm that deletion of *S*. *suis* serotype 2 CPS biosynthesis genes results in non-encapsulation of mutant strains, regardless of strain background.

### Role of capsular polysaccharide in resistance of *S*. *suis* serotype 2 to phagocytosis by dendritic cells is strain-dependent

**Fig 3:**
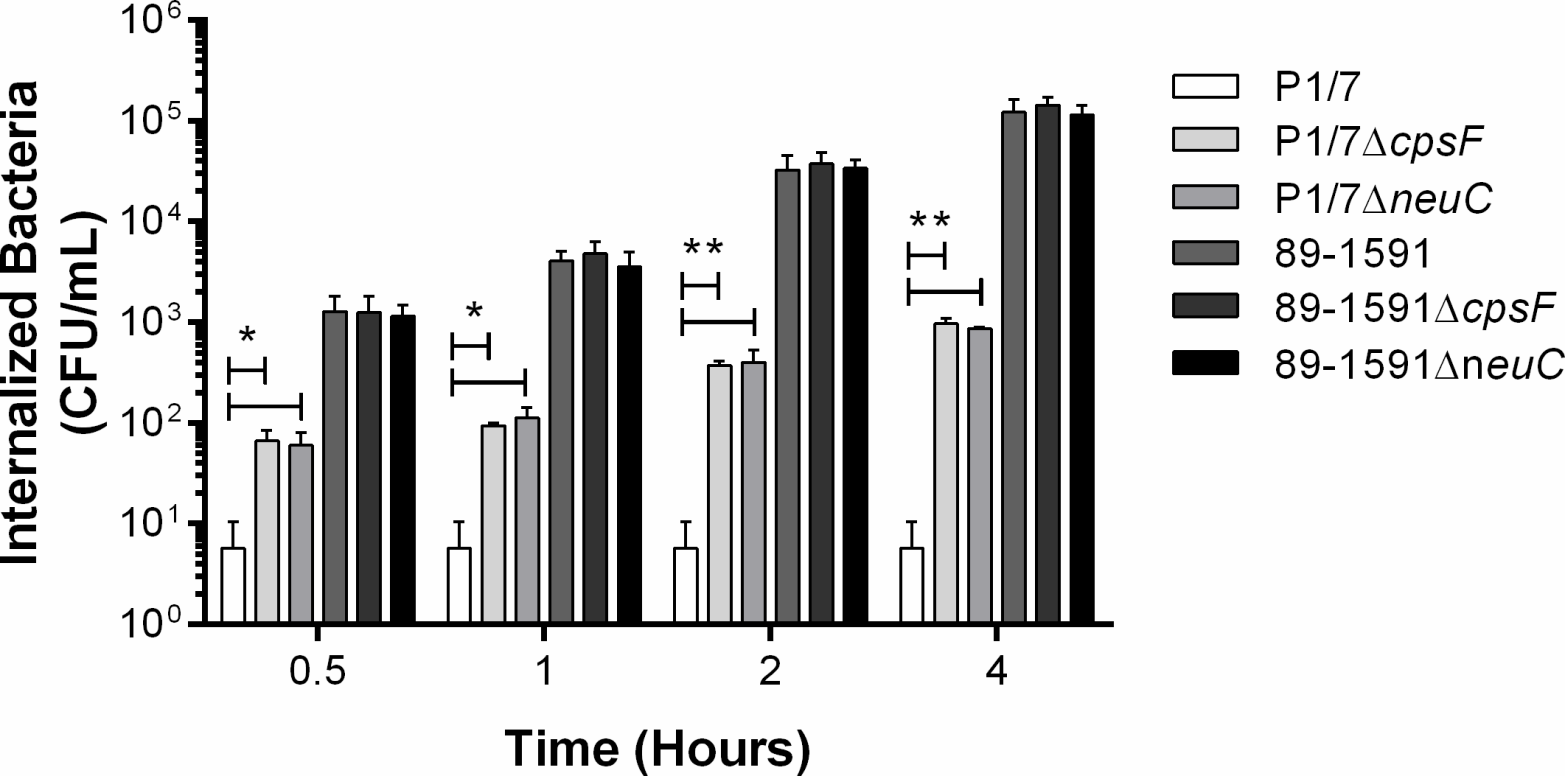
Strain-dependent role of the capsular polysaccharide in *S*. *suis* serotype 2 resistance to phagocytosis by dendritic cells (DCs). Internalization kinetics (0.5 to 4 h) of wild-type and non-encapsulated mutant strains by DCs. Data represent the mean ± SEM from four independent experiments. * (*p* < 0.05) and ** (*p* < 0.01) indicate a significant difference between P1/7 and P1/*7*Δ*cpsF* or P1/*7*Δ*neuC*.

Resistance to internalization by phagocytes is an important property conferred to *S*. *suis* serotype 2 by presence of its CPS [22]. To evaluate this function, DCs from C57BL/6 mice were used. This breed of mice has been largely used in the literature [16, 22, 32] since it is inbred, which greatly reduces variability between individuals and the number of animals required. Moreover, results obtained using DCs from C57BL/6 mice have been demonstrated to be representative of porcine and human DCs [35, 36]. As previously described, the ST1 strain P1/7 was little internalized by DCs (less than 10 CFU/mL), with internalization levels remaining constant over time (Fig 3). By contrast, non-encapsulated mutants P1/7Δ*cpsF* and P1/7Δ*neuC* were significantly more internalized (*p* < 0.05), with levels of internalized bacteria increasing with time (Fig 3). Meanwhile, the ST25 strain 89–1591 was significantly more internalized than the ST1 wild-type strain P1/7 (*p* < 0.01), and this regardless of the incubation time (Fig 3). Surprisingly, no differences were observed between internalization levels of the ST25 strain 89–1591 and its non-encapsulated mutants, 89–1591Δ*cpsF* and 89–1591Δ*neuC*, by murine DCs (Fig 3). Lack of difference between the ST25 strain 89–1591 and its non-encapsulated mutants was not due to low or non-encapsulation of the wild-type strain in cell culture medium as determined by hydrophobicity assay following infection of DCs (data not shown).

### Interference of dendritic cell cytokine production by presence of the *S*. *suis* serotype 2 capsular polysaccharide is strain-dependent

**Fig 4:**
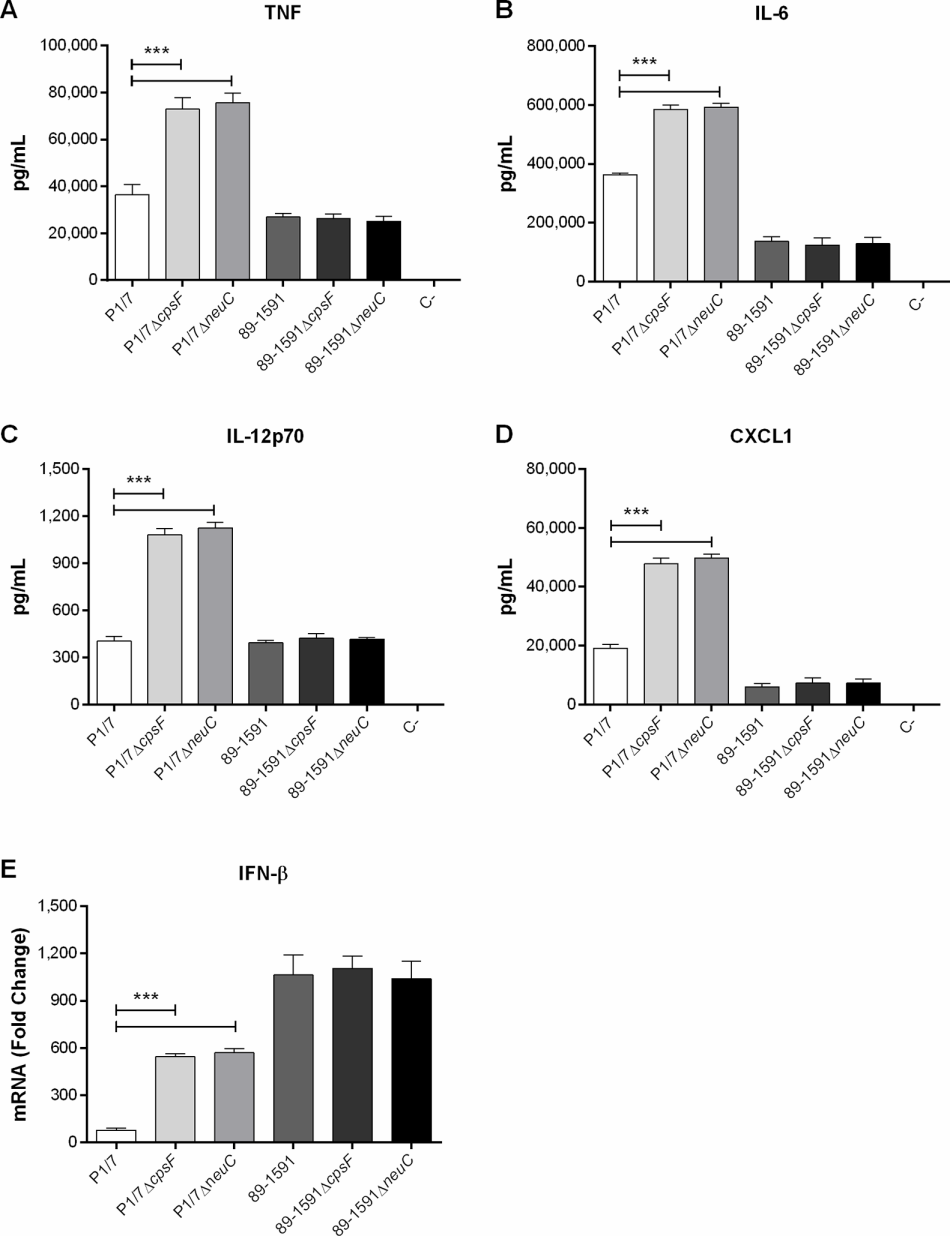
Strain-dependent role of capsular polysaccharide in interference of *S*. *suis* serotype 2-induced cytokine production by dendritic cells (DCs). Cytokine production by DCs following infection with wild-type and non-encapsulated mutant strains after 16 h of incubation, as measured by ELISA, with the exception of IFN-β, after 6 h of incubation, by RT-qPCR. Production of TNF (A), IL-6 (B), IL-12p70 (C), CXCL1 (D), and IFN-β (E). Data represent the mean ± SEM from four independent experiments. C- denotes the negative control (cells in medium alone). *** (*p* < 0.001) indicates a significant difference between P1/7 and P1/*7*Δ*cpsF* or P1/*7*Δ*neuC*.

Presence of the *S*. *suis* serotype 2 CPS has also been associated with interference of cytokine production by myeloid cells, including DCs [16, 17]. While the ST1 strain P1/7 and the ST25 strain 89-1591 both induced a variety of cytokines following infection of murine DCs (TNF, IL-6, IL-12p70, and CXCL1), only levels of IL-6 and CXCL1 induced by P1/7 (ST1) were significantly higher than those induced by 89–1591 (ST25) (*p* < 0.01) (Fig 4). As previously reported [16], absence of CPS from strain P1/7 (ST1) significantly increased production of TNF, IL-6, IL-12p70, and CXCL1 (*p* < 0.001) by DCs (Fig 4A–4D). In contrast, absence of CPS did not affect production of these mediators by DCs following infection with the ST25 strain 89–1591 (Fig 4A–4D). Once again, lack of difference between the ST25 strain 89–1591 and its non-encapsulated mutants was not due to low or non-encapsulation of the wild-type strain in cell culture medium as determined by hydrophobicity assay following infection of DCs (data not shown).

We recently demonstrated a mechanism complementary to surface-associated receptor activation whereby internalization of *S*. *suis* can lead to induction of IFN-β by DCs [32]. Since internalization levels of the 89–1591 non-encapsulated mutants were similar to those of the wild-type strain, it was hypothesized that IFN-β expression might be similar between strain 89–1591 and its non-encapsulated mutants. As previously described, the ST25 strain 89–1591 induced significantly higher levels of IFN-β expression from DCs than did the ST1 strain P1/7 6 h p.i. (*p <* 0.001) (Fig 4E) [32]. Both non-encapsulated mutants of the ST1 strain P1/7 induced significantly higher levels of IFN-β expression than did the wild-type strain (*p* < 0.001) (Fig 4E). By contrast, IFN-β expression was similar between 89–1591 and its two mutants (Fig 4E**)**.

### Capsular polysaccharide is required for *S*. *suis* serotype 2 whole blood bactericidal resistance regardless of strain background

**Fig 5:**
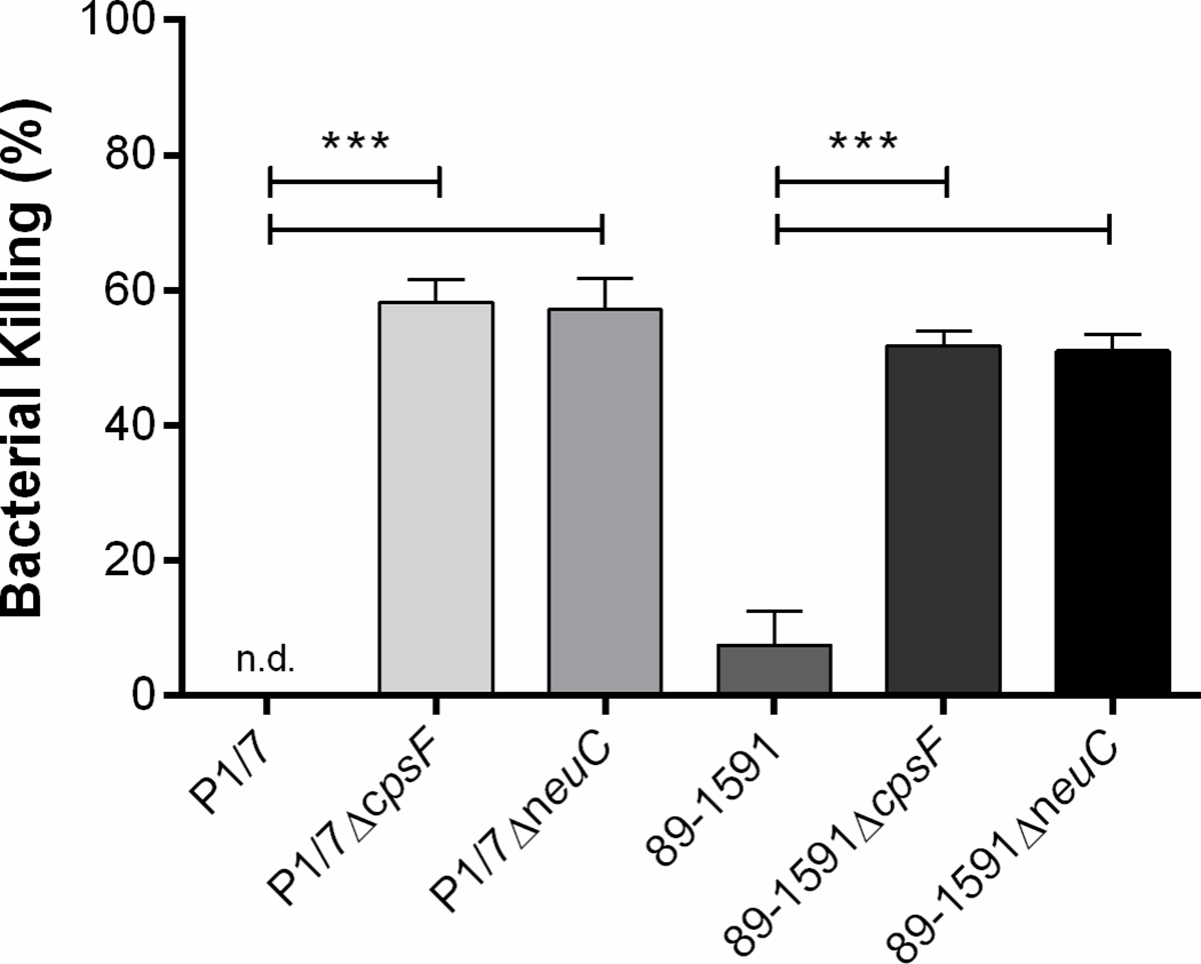
The *S*. *suis* serotype 2 capsular polysaccharide is required for resisting the bactericidal effect of whole blood regardless of strain background. Capacity of wild-type strains and non-encapsulated mutants to resist the bactericidal effect of murine whole blood after 4 h of incubation. Percentage of bacterial killing was calculated in comparison to bacteria in plasma alone. Data represent the mean ± SEM from four independent experiments. n.d. denotes not detected. *** (*p* < 0.001) indicates a significant difference between wild-type strains (P1/7 or 89–1591) and their mutants (Δ*cpsF* or Δ*neuC*).

While presence of the North American ST25 CPS only minimally modulated interactions with murine DCs *in vitro*, its role in a context more reflective of the *in vivo* situation remained unknown. Consequently, the role of the presence of the ST25 strain CPS in resisting the bactericidal effect of murine whole blood was evaluated. As previously reported [4], the ST1 strain P1/7 completely resisted killing by whole blood, while the ST25 strain 89–1591 was somewhat less resistant (Fig 5). The non-encapsulated mutants of strain P1/7 were significantly less resistant to the bactericidal effect of murine whole blood than their wild-type strain (*p* < 0.001), with nearly 60% of bacteria killed after 4 h of incubation (Fig 5). Similarly, non-encapsulation of strain 89–1591 resulted in significantly greater killing by whole blood (*p* < 0.001), with levels of killed bacteria similar to those of P1/7 non-encapsulated mutants (Fig 5).

### Capsular polysaccharide is required for virulence of the *S*. *suis* serotype 2 ST25 strain 89–1591 in a mouse model of infection

**Fig 6:**
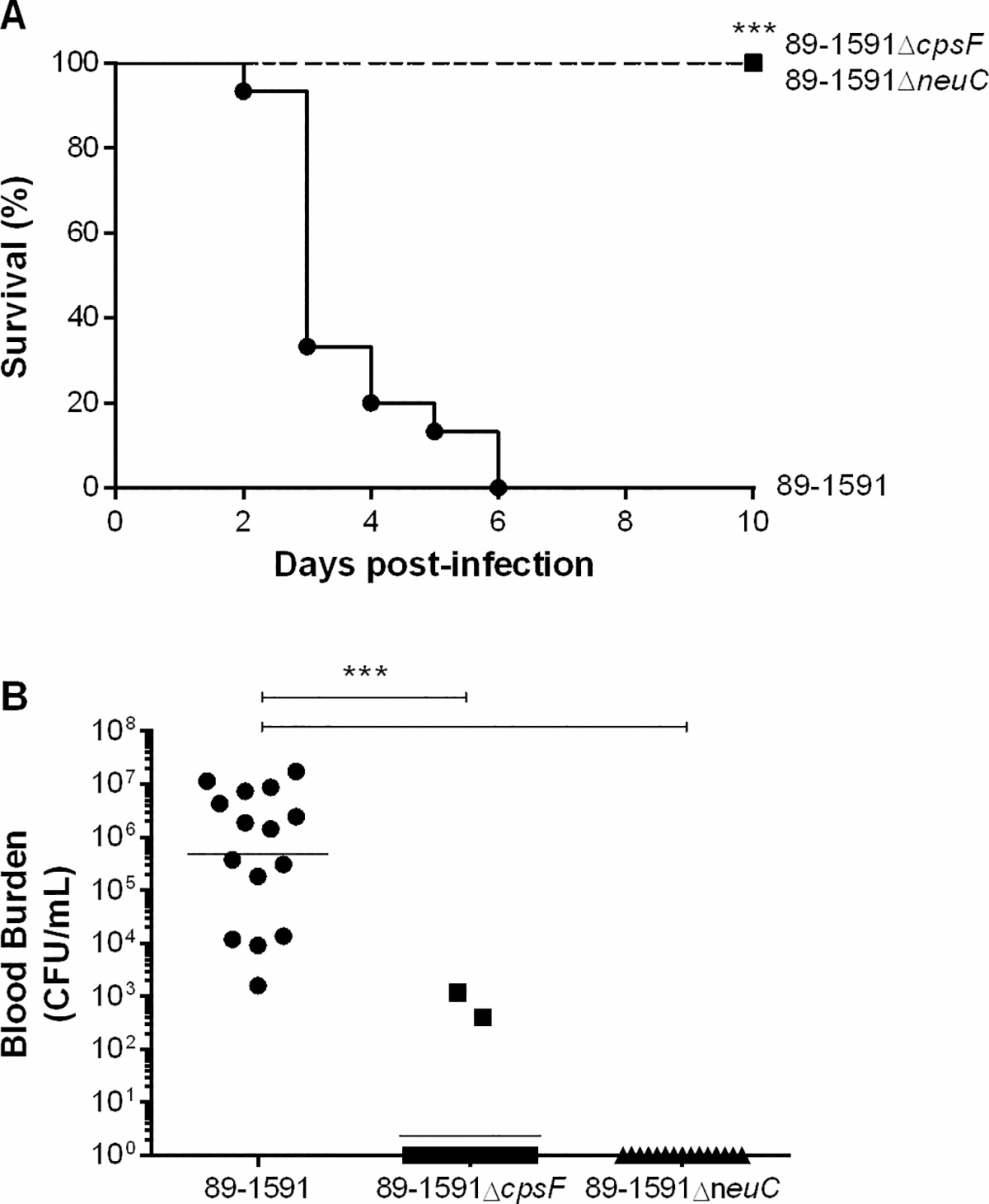
Capsular polysaccharide is required for virulence of the *S*. *suis* serotype 2 ST25 strain 89–1591 in a mouse model of infection and persistence in blood. Survival (A) and blood bacterial burden 24 h post-infection (B) of CD-1 mice following intraperitoneal inoculation of 5 x 10^7^ CFU of wild-type strain 89–1591 or its non-encapsulated mutants 89-1591Δ*cpsF* and 89-1591Δ*neuC*. Data represent the survival curves (A) or geometric mean (B) of 15 mice/strain. *** (*p* < 0.001) indicates a significant difference between survival or blood bacterial burden of mice infected with wild-type strain 89–1591 and those infected with non-encapsulated mutants (89-1591Δ*cpsF* and 89–1591Δ*neuC*).

To evaluate the role of the North American ST25 CPS in virulence and development of clinical disease, a well-developed CD-1 mouse model of infection was used in which bacteria were inoculated via the intraperitoneal route [9, 23, 37–39]. As previously reported, the ST25 wild-type strain 89–1591 induced disease in mice, with 100% of mice succumbing within six days of infection (Fig 6A) [4, 9]. While some mice succumbed to septic shock, most mice developed clinical signs of meningitis, which is a characteristic of this strain. By contrast, none of the mice infected with either of its non-encapsulated mutants (89–1591Δ*cpsF* or 89-1591Δ*neuC*) succumbed to disease (*p* < 0.001) (Fig 6A). In fact, these mice only developed transient signs of infection such as rough coat hair following inoculation of bacteria. Viability of the inoculum was verified prior to and after infection with no differences between (data not shown).

In order to explain these differences in virulence, blood bacterial burden was evaluated 24 h p.i., time at which only bacteria capable of persisting are detected. All mice infected with the wild-type strain 89–1591 presented blood bacterial burdens that averaged 5 x 10^5^ CFU/mL (Fig 6B). On the other hand, almost no bacterial burden was detected in blood of mice infected with either of the non-encapsulated mutants, levels of which were significantly lower than those of mice infected with the wild-type strain (*p* < 0.001) (Fig 6B).

## Discussion

*S*. *suis* serotype 2 is an important porcine bacterial pathogen and zoonotic agent responsible for sudden death, septic shock, and meningitis [1]. Of its different virulence factors, the CPS is considered one of the only “truly” critical virulence factors, being associated with anti-phagocytic properties, protection against bacterial killing, and a capacity to interfere with cytokine production [10]. However, previous studies have used Eurasian strains (virulent ST1 and ST7), which greatly differ from their North American counterparts, including intermediate virulent ST25 strains [10].

Biosynthesis of the *S*. *suis* serotype 2 CPS involves a variety of genes encoded by the *cps* locus of which mutants deficient for *cpsB*, *cpsE*, *cpsF*, *cpsG*, *cpsJ*, *cpsL*, *neuB*, and *neuC* have been created, all using Eurasian ST1 strains or the Chinese ST7 strain [16, 18, 19, 34, 40]. In accordance with these studies, deletion of *cpsF* or *neuC* genes also caused non-encapsulation of the ST25 strain 89–1591, which suggests that deletion of a gene from the serotype 2 *cps* locus probably causes non-encapsulation regardless of strain background. A similar phenomenon was observed for *S*. *suis* serotype 14, whereby deletion of the *cpsB* or *neuC* genes caused a loss of CPS [41]. Based on current knowledge obtained with the serotypes 2 and 14 only, it may be hypothesized that tampering of the *S*. *suis cps* locus results in non-encapsulation independently of serotype. However, given the structural similarities between serotypes 2 and 14, including presence of sialic acid, the use of more divergent serotypes will be required to confirm this hypothesis [42–44].

Previous studies with Eurasian strains have demonstrated that serotype 2 CPS is a critical anti-phagocytic factor that protects against internalization by DCs (as well as other phagocytes) of murine, porcine, and human origin [13, 16, 35, 36, 45]. However, this property was not observed herein using a ST25 strain: as such, it would be interesting to confirm using other cell types. It is important to note that composition and structure are responsible for serotype determination [42, 46], and that all serotype 2 strains have the same CPS antigenically, which indicates a highly similar structure and composition. In addition, the *cps* locus is identical in all serotype 2 strains sequenced to date, regardless of sequence type [47]. Moreover, thickness of CPS was determined to be similar between strains P1/7 and 89–1591 by electron microscopy [32]. As such, though results are currently unavailable, it can be hypothesized that differences in internalization might be due to differential exposition and/or composition of the underlying surface components. Unfortunately, identity of all sub-capsular *S*. *suis* serotype 2 components remains unknown, and future studies will be necessary to both confirm this hypothesis and identify differences between strains. Interestingly, Houde *et al*. demonstrated that presence of serotype 2 CPS destabilizes host cell lipid microdomains, thus preventing lactosylceramide accumulation at the cell membrane, which blocks *S*. *suis* phagocytosis and lactosylceramide-dependent recognition of *S*. *suis* [48]. Although lactosylceramide-dependent recognition of *S*. *suis* appears to be important for internalization, the bacterial component recognized remains unknown, though it is supposed to be located at the bacterial surface [48]. Since the ST25 strain 89–1591 was more internalized by DCs, regardless of it being well-encapsulated, it may be hypothesized that expression, surface distribution, and/or exposition/masking of the yet unknown *S*. *suis* component recognized by lactosylceramide might vary between the two strains.

This differential role of the CPS regarding its anti-phagocytic properties suggests a potential paradigm shift. In fact, certain misconceptions regarding the *S*. *suis* CPS are commonplace: while CPS protects by masking surface components, many of which are important immune activators, it is not impenetrable, rather being analogous to a net. Indeed, the correct term regarding the serotype 2 CPS structure is random coil, indicating that it is constantly shifting over the entirety of the bacterium, being fluid rather than static [42]. Moreover, *S*. *suis* serotype 2 possesses the ability to modulate presence of its CPS, whose thickness might depend on the environment faced [20]. Finally, data suggest that unlike Group B *Streptococcus*, the *S*. *suis* serotype 2 CPS is not firmly attached to its surface, as it is easily recovered following autoclave treatment [42]. Consequently, though the CPS surrounds *S*. *suis* and protects it from recognition by phagocytes, it does not necessarily mask all surface components simultaneously.

While phagocytosis assays are important to better understand the interactions between *S*. *suis* and phagocytic cells such as DCs, they do not necessary represent the complex reality of the host. Moreover, clearance of *S*. *suis* by phagocytes depends not only on its internalization, but most importantly on its killing, which *S*. *suis* attempts to thwart in order to survive and induce disease [10]. In fact, the anti-phagocytic role of the CPS has long been associated with survival of the pathogen in blood via resistance to bacterial killing [10]. Unlike results obtained with DCs alone, presence of CPS was required for resisting the bactericidal effect of murine blood regardless of strain background. Differently from phagocytosis assays, whole blood is composed of a variety of immune cell types, of which neutrophils and monocytes predominate; while dendritic cells are important phagocytes, they are more commonly distributed throughout tissues rather than in blood [21, 49]. Furthermore, the phagocytic and killing capacity of immune cells differs: while DCs were reported to internalize *S*. *suis* more efficiently than monocytes, it was also demonstrated that neutrophils, which constitute the most important subset of blood phagocytes, are the most efficient at killing *S*. *suis* [13, 45]. Differences between results obtained using the phagocytosis assay with DCs alone and whole blood bactericidal assay confirm the lack of correlation between resistance to phagocytosis by a single cell type and resistance to killing in a complex model reflective of the systemic compartment [4]. Though phagocytosis assays are crucial for dissecting the cell-pathogen interactions, conclusions should be carefully interpreted and may not always be extrapolated to the more complex systems required to better comprehend the interplay *in vivo*.

Recognition of *S*. *suis* by various cell receptors results in production of cytokines, which participate in the inflammatory response required to clear the pathogen [22, 23]. Interference of cytokine production by myeloid cells is thus an important tactic used by *S*. *suis* to avoid detection by host cells and in which presence of the serotype 2 CPS participates. While absence of CPS from strain P1/7 (ST1) resulted in increased cytokine production due to recognition of surface components by host receptors, as previously reported with other myeloid cells [16, 17, 35, 36, 50, 51], the lack of differences between the well-encapsulated ST25 wild-type strain 89–1591 and its non-encapsulated mutants is novel. Interestingly, the ST1 strain P1/7 induced higher levels of IL-6 and CXCL1, but not of TNF and IL-12p70, than the ST25 strain 89–1591. This result, while unexpected, is difficult to explain based on current knowledge since very little studies have used North American ST25 strains. It was previously demonstrated that plasma levels of TNF, IL-6, and CXCL1, but not IL-12p70, are higher during systemic infection in mice infected with strain P1/7 than those infected with strain 89–1591 [4]. *In vitro*, however, a collection of North American and European strains induced similar mRNA levels of TNF, IL-6, and IL-8 from porcine whole blood [52], while another virulent European serotype 2 ST1 strain (reference strain 735) induced higher levels of IL-6, but not IL-8, from human brain microvascular endothelial cells [53]. Once again, these differences in cytokine production might be due to differential activation resulting from varying exposition and/or composition of underlying immunostimulatory surface components. Finally, given that these cytokines were generally more induced by strain P1/7 than 89–1591 *in vivo*, but not necessarily with DCs, indicates that other cell types are probably involved in inflammatory mediator production *in vivo*, including monocytes, neutrophils, and/or Natural Killer cells. Together though, the current lack of knowledge justifies the need in studying strains from different genetic backgrounds other than ST1 and ST7, but also with other immune cell types, in order to better understand the complex pathogen that is *S*. *suis*.

Since *S*. *suis* is a classical extracellular pathogen, activation of surface-associated receptors, including Toll-like receptors (TLRs), is crucial for cytokine production [17, 22]. As such, the differential role of the serotype 2 CPS between strains P1/7 and 89–1591 might be due to differential exposition and/or composition of surface components. Though identity of the surface components involved is unknown, potential candidates include lipoproteins, which are immunostimulatory proteins whose composition is highly variable between bacterial species and possibly even between strains [54]. Alongside phagocytosis-independent surface-associated cell receptor-dependent cytokine production, we recently demonstrated a new complementary mechanism whereby, following phagocytosis, the *S*. *suis* nucleic acids activate the endosomal TLR7 and TLR9, leading to production of IFN-β [32]. Differential results in IFN-β induction by strain P1/7 and its mutants, in contrast to strain 89–1591, illustrate how production of internalization-dependent cytokines correlates with phagocytosis levels.

The contrasting interactions between DCs and the *S*. *suis* serotype 2 strains P1/7 and 89-1591 *in vitro* suggested potentially differential roles of their CPS in virulence. Absence of CPS was previously reported to result in avirulence, regardless of the *cps* gene mutated in Eurasian ST1 or ST7 strains [18, 19, 34]. In accordance, yet in contrast to interactions with DCs alone, non-encapsulation of the ST25 strain also resulted in avirulence, due to an inability of the mutants to persist in the bloodstream. This inability is in accordance with whole blood killing results, confirming that this test is a good correlate of virulence [4].

Interestingly, while lack of serotype 2 CPS resulted in similar or increased cytokine production *in vitro* depending on the strain, these non-encapsulated strains are avirulent *in vivo*. Though *S*. *suis*-induced exacerbated inflammation is a hallmark of disease [10], the lack of resistance of non-encapsulated mutants to the bactericidal effect of whole blood results in their rapid clearance and overall reduced inflammatory activation of host cells *in vivo*. By contrast, resistance of well-encapsulated *S*. *suis* serotype 2 strains to killing by blood leukocytes favors their replication, persistence, stimulation, and activation of host cells, resulting in an exacerbated inflammation responsible for clinical disease and host death [4, 23].

In conclusion, this study is the first to demonstrate that while the *S*. *suis* serotype 2 CPS remains critical for persistence in blood, development of clinical disease, and overall virulence, its role in the interactions with immune cells is strain-specific. In fact, the CPS has been classically associated with anti-phagocytic properties and masking of bacterial surface components. However, these properties are not necessarily applicable to all *S*. *suis* strains. This shift in paradigm suggests that while certain factors may be important for virulence of *S*. *suis* serotype 2, strain background could be as determining [55]. As such, further studies using additional ST25 strains, as well as strains from other STs and of varying virulence, will be necessary to confirm these results as being strain-specific, ST-specific or other. Importantly, this study reiterates that the use of strains from varying backgrounds is required in order to better characterize the *S*. *suis* pathogenesis and that generalized conclusions should avoided.

## Supporting information

S1 Appendix Evaluation of clinical signs and scoring following intraperitoneal injection of *Streptococcus suis* serotype 2 in mice. (PDF)
