# Supplementary material for: Role of the Streptococcus suis serotype 2 capsular polysaccharide in the interactions with dendritic cells is strain-dependent but remains critical for virulence
Source: PLoS One. 2018 Jul 12;13(7):e0200453. doi: 10.1371/journal.pone.0200453 (PMC6042740; doi:10.1371/journal.pone.0200453)
Supplement: S1 Appendix — (PDF) [file pone.0200453.s001.pdf]

Supporting Information – S1 Appendix

Evaluation of clinical signs and scoring following intraperitoneal injection of *Streptococcus suis* serotype 2 in mice

1) Without opening the cage, evaluate the behavior of each mouse.
2) Open the cage and examine the behavior of each mouse in more detail without manipulating.
3) Evaluate the clinical signs of each mouse according to the table below and inscribe the clinical score associated with each clinical sign.

Clinical signs in mice following intraperitoneal injection of *S. suis* serotype 2

Code	Clinical Sign	Score
**A**	Normal behavior	0
**B**	Rough hair coat	1
**C**	Swollen/closed eyes	1
**D**	Ocular edema	1
**E**	Prostration	1
**F**	Depression	1
**G**	Transitory difficulty breathing	1
**H**	Lethargy	7
**I**	Meningitis	7
**J**	Frenetic jumping	7
**K**	Pedaling	7
**L**	Sudden excitement followed by death	7
**M**	Weight loss greater than 20% of the initial weight	5
**N**	Death	10

Clinical signs associated with meningitis

i	Spatial disorientation
ii	Hyperexcitation/opisthotonos
iii	Circular walking with head tilt
iv	Sudden excitement followed by recumbency
v	Clonic movement

4) Weigh each mouse daily.
5) Tabulate the clinical score associated with each mouse and determine the action required according to the following table:

Score	Health Status	Action Required
0	Clinically normal	None
1-3	Post-infection reaction (transitory)	None
4-5	Moderately sick	Re-evaluate every 4 h
6	Visibly sick	Re-evaluate every 3 h; if no improvement after 24 h, euthanasia is required
7 or more	Extremely sick	Immediate euthanasia required

6) Euthanasia is carried out using CO_2 followed by cervical dislocation.
